# Transforming assessment of speech in children with cleft palate via online crowdsourcing

**DOI:** 10.1371/journal.pone.0227686

**Published:** 2020-01-09

**Authors:** Anne M. Sescleifer, Caitlin A. Francoisse, Janna C. Webber, Jeffrey D. Rector, Alexander Y. Lin

**Affiliations:** 1 Division of Plastic Surgery, Saint Louis University School of Medicine, Saint Louis, Missouri, United States of America; 2 St. Louis Cleft-Craniofacial Center, Division of Pediatric Plastic Surgery, SSM Health Cardinal Glennon Children’s Hospital at SLU, Saint Louis, Missouri, United States of America; 3 Rector Consulting, San Francisco, California, United States of America; Ohio State University, UNITED STATES

## Abstract

**Objective:**

Speech intelligibility is fundamental to social interactions and a critical surgical outcome in patients with cleft palate. Online crowdsourcing is a burgeoning technology, with potential to mitigate the burden of limited accessibility to speech-language-pathologists (SLPs). This pilot study investigates the concordance of online crowdsourced evaluations of hypernasality with SLP ratings of children with cleft palate.

**Methods:**

Six audio-phrases each from children with cleft palate were assessed by online crowdsourcing using Amazon Mechanical Turk (MTurk), and compared to SLP’s gold-standard hypernasality score on the Pittsburgh Weighted Speech Score (PWSS). Phrases were presented to MTurk crowdsourced lay-raters to assess hypernasality on a Likert scale analogous to the PWSS. The survey included clickable reference audio samples for different levels of hypernasality.

**Results:**

1,088 unique online crowdsourced speech ratings were collected on 16 sentences of 3 children with cleft palate aged 4–8 years, with audio averaging 6.5 years follow-up after cleft palate surgery. Patient 1 crowd-mean was 2.62 (SLP rated 2–3); Patient 2 crowd-mean 2.66 (SLP rated 3); and Patient 3 crowd-mean 1.76 (SLP rated 2). Rounded for consistency with PWSS scale, all patients matched SLP ratings. Different sentences had different accuracies compared to the SLP gold standard scores.

**Conclusion:**

Online crowdsourced ratings of hypernasal speech in children with cleft palate were concordant with SLP ratings, predicting SLP scores in all 3 patients. This novel technology has potential for translation in clinical speech assessments, and may serve as a valuable screening tool for non-experts to identify children requiring further assessment and intervention by a qualified speech language pathology expert.

## Introduction

Cleft lip-nose-palate is the most common birth defect requiring surgical intervention in the United States, occurring in 1 out of every 600 live births [1,2]. Aberrant anatomy allows for increased airflow through the nasal cavity during speech, resulting in an atypical speech resonance termed hypernasality. Despite the frequency with which cleft repair surgeries are performed, objective evaluation of surgical outcomes including degree of speech impairment remains a challenge since plastic surgery aims to restore human features that are inherently subjective and difficult to quantify.

Speech language pathologists (SLPs) are experts trained to assess and treat speech disorders, and act as integral members of the cleft team to monitor post-operative speech outcomes [3]. Up to 30% of patients with primary palate repairs have unsatisfactory speech results and require revision procedures [4]. SLPs help identify these patients for early intervention; however, there is a nationwide shortage of SLPs, especially in low income or rural areas [5]. The high cost and limited access to speech experts can pose a barrier to care, and presents an immediate need for speech evaluations that are rapid, consistent, low-cost, and widely accessible to patients with cleft from all backgrounds.

Online crowdsourcing platforms, such as Amazon Mechanical Turk (MTurk), offer a potential solution to this barrier. Crowdsourcing refers to large-scale online data collection from lay raters. Amazon’s MTurk, an online marketplace, allows clinicians or researchers to post a task in exchange for small online payments. This novel technology is revolutionizing research by aggregating large numbers of laypeople evaluations to approximate expert opinion. Previous studies suggest that MTurk is an effective tool for the evaluation of perceptual speech outcomes, providing high-quality and consistent assessments in a rapid, cost-effective manner [6].

This novel study represents the first time that speech samples from children with cleft palate have been assessed using online crowdsourcing. The main aim of this pilot study is to investigate the validity of online crowdsourcing as an assessment measure in the evaluation of hypernasal speech.

## Methods

This study was approved by the Saint Louis University School of Medicine Institutional Review Board (Protocol #28839). Written consent was obtained. Our prospective, case-series study was conducted at the St. Louis Cleft-Craniofacial Center at SSM Health Cardinal Glennon Children’s Hospital at Saint Louis University.

Speech clips were sampled from indicated videonasendoscopy (VNE) recordings in children with cleft palate, recorded at previous routine cleft team clinic visits. Audio recordings were extracted from children’s VNEs using QuickTime Player (Apple, Inc., Cupertino, CA). These recordings had been previously assessed by the cleft team's SLP in clinic and rated for hypernasality. The same SLP rated the audio-only samples prior to publication of speech on MTurk, for intra-rater reliability. Samples included were screened for velopharyngeal mislearning and compensatory strategies, and place of articulation was accurate for the targeted phoneme. Test sentences were selected for frequency with which they appeared in clinical samples, as well as for variation in sound focus (phoneme). Each test phrase emphasized a unique phoneme, which was repeated three times throughout the sentence. These sounds could be categorized by speech category, including “plosives” which require a buildup of air to correctly articulate the sound, and “fricatives” which require the friction of breath in a narrow opening. Six main phrases were used: “Katie likes cookies” (KC), “Tell Ted to try” (TT), “Peter has a puppy” (PP), “Should I wash the dishes” (SW), “Sissy sissy sissy” (SS), and “Zippers are easy to close” (ZC) [Table 1].

**Table 1:** Test phrases. Each test phrase was selected for a unique sound focus, which was repeated three times in the sentence. These sounds could be categorized by speech category, including “plosives” which require a buildup of air to correctly articulate the sound, and “fricatives” which require the friction of breath in a narrow opening.

Code	Sentence	Sound Focus	Speech Category
**KC**	**K**atie li**k**es coo**k**ies.	“k”	Plosive (velar)
**TT**	**T**ell **T**ed to **t**ry.	“t”	Plosive (alveolar)
**PP**	**P**eter has a **p**u**pp**y.	“p”	Plosive (bilabial)
**SW**	**Sh**ould I wa**sh** the di**sh**es?	“sh”	Fricative (postalveolar)
**SS**	**S**i**ss**y, **s**i**ss**y, **s**i**ss**y	“s”	Fricative (alveolar)
**ZC**	**Z**ippers are ea**sy** to clo**se**.	“z”	Fricative (alveolar)

These VNE-extracted audio phrases were presented in survey format to internet raters recruited from the online crowdsourcing platform Amazon Mechanical Turk (MTurk). Both SLPs and crowdsourced lay raters assigned perceptual speech scores based on the hypernasality component of the Pittsburgh Weighted Speech Score (PWSS). The PWSS scale ranges from 0 to 4, based on the rater’s interpretation of speech hypernasality (Table 2) [7]. Typically, SLPs assign whole-number ratings, but may also assign a range; for example, 2–3, if they perceive the hypernasality to be “mild-moderate” that straddle two perceptual grades. Score ranges were converted to a single average number (in this example 2.5) for numerical analysis.

**Table 2:** Pittsburgh Weighted Speech Score (PWSS). PWSS Hypernasality Component was used to rate speech samples, where 0 corresponds to no hypernasality and 4 corresponds to severe hypernasality.

Pittsburgh Weighted Speech Score Hypernasality Component	
*Score*	*Rating*
0	No hypernasality
1	Minimal hypernasality
2	Mild hypernasality
3	Moderate hypernasality
4	Severe hypernasality

MTurk survey participants were prompted with the following screen instructions: “Please listen to this sound sample and focus on the child’s voice repeating the instructor’s voice. Which of the following standard samples of hypernasality do you think the child’s speech sounds closest to?” Sound clips were rated on a 5-point Likert scale, corresponding to the hypernasal component (0–4) of the PWSS. The survey page had three clickable "gold standard" samples available for the rater to use as reference, representing PWSS hypernasality of 0 (none), 2 (mild), and 4 (severe). These standards were determined by an SLP with extensive experience evaluating speech of children with cleft palate, who routinely provides clinical speech assessments in our weekly cleft team clinic, where our center is an affiliated team of the American Cleft Palate-Craniofacial Association (ACPA). To enhance the quality of crowdsourced data, MTurk participants were required to have the MasterWorkers qualification, which distinguishes top workers identified by MTurk for consistent and high-quality work. Our study employed the additional quality checks of: USA-based IP addresses; native language English, and no self-reported personal history of hearing or speech problems.

We investigated two primary outcomes: (1) Do averaged MTurk ratings align with SLP gold standard ratings?, and (2) Are some phrases better than others at predicting hypernasality ratings in online assessments? MTurk ratings for each patient were averaged, to compare to that patient’s gold standard SLP-rated speech score. Given the PWSS is a whole number scale, an acceptable margin of accuracy in this study was set at 0.5 when comparing MTurk averaged results to SLP gold standard ratings. To determine whether some phrases were more accurate predictors of hypernasality scores, ANOVA with Tukey post-hoc pairwise comparisons was conducted. Results are reported in averages ± standard error.

## Results

Speech samples were provided by three children with history of cleft palate, ages 4–8 years old, with timing of recordings ranging from pre-surgical repair to 6.5 years follow-up. Surveys containing 16 unique speech samples (5–6 sentences per child) were posted to MTurk, and 68 MTurk participants provided survey responses. Each MTurk user provided a single rating for each of the samples, totaling 1,088 unique speech ratings.

MTurk crowdsourced ratings were compared to SLP gold standard ratings. When all MTurk ratings were averaged, Patient 1 crowd mean was 2.62 with standard error of mean 0.059 (SLP rated 2–3); Patient 2 crowd mean was 2.66 ±0.060 (SLP rated 3); and Patient 3 crowd mean was 1.76 ±0.070 (SLP rated 2). When compared to the SLP rating, each MTurk averaged score was within our margin of accuracy of within 0.5 from the gold standard (Table 3).

**Table 3:** Patient Demographics. Patient demographics, including age at initial palatoplasty surgery, time elapsed from surgery to speech sample, and age at speech sample recording are presented. The SLP hypernasality sub-score of the Pittsburgh Weighted Speech Score is reported.

Child	Gender	Age at Surgery	Type of Surgery	Time since Surgery	Age at Recording	SLP Score
P1	Female	1 year	Primary Palatoplasty	2 years	4 years	2–3
P2	Male	1 year	Primary Palatoplasty	7 years	8 years	3
P3	Male	N/A	N/A	Pre-op	5 years	2

**Fig 1:**
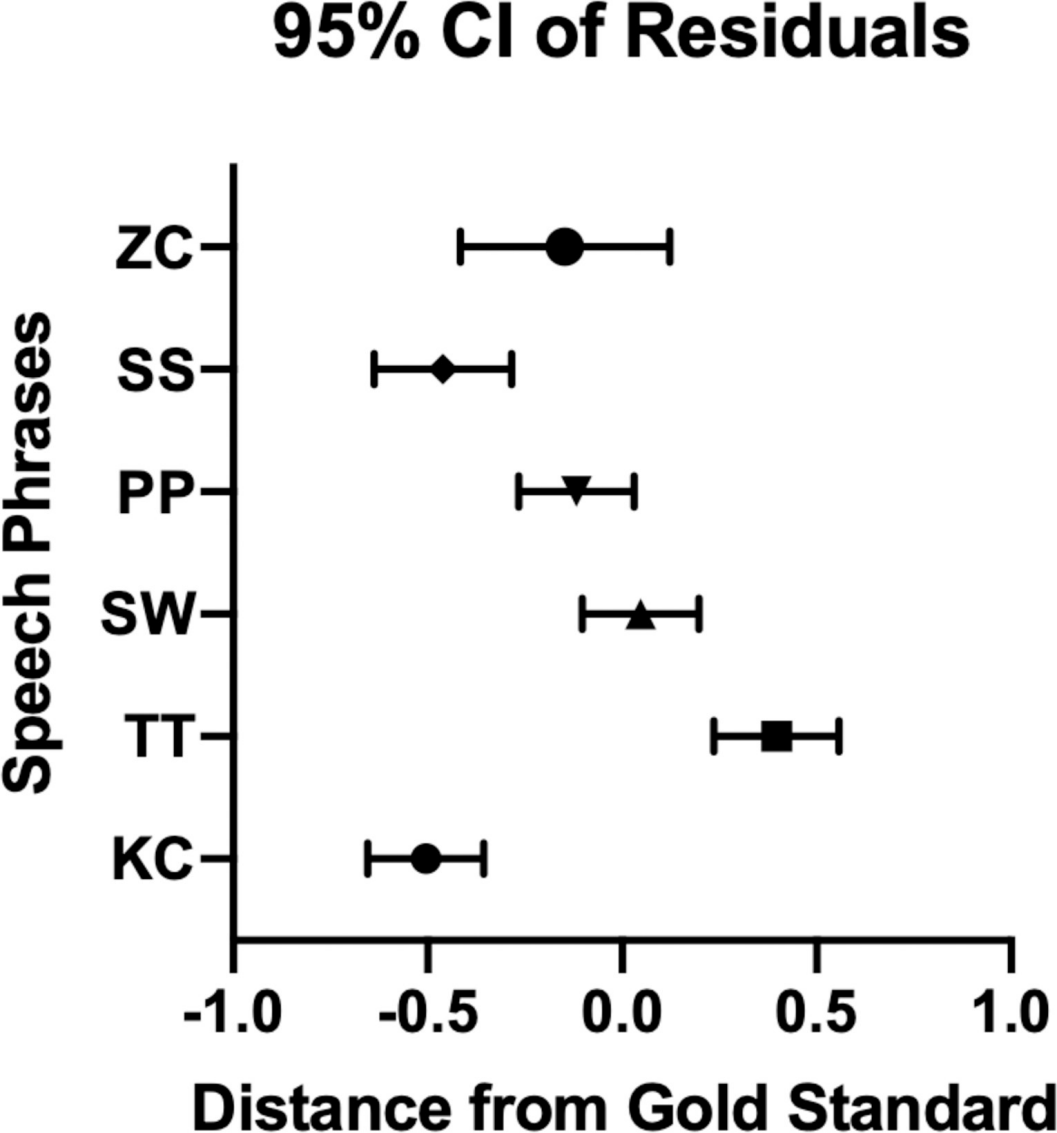
95% Confidence Interval (CI) of Residuals. The 95% CIs were calculated based on the residuals (MTurk crowdsourced mean–SLP gold standard score) for each phrase. The phrases ZC, PP, and SW crossed 0.0, while the phrases SS, TT, and KC did not. This suggests that ZC, PP, and SW crowdsourced MTurk speech ratings are not significantly different from the SLP expert score.

**Fig 2:**
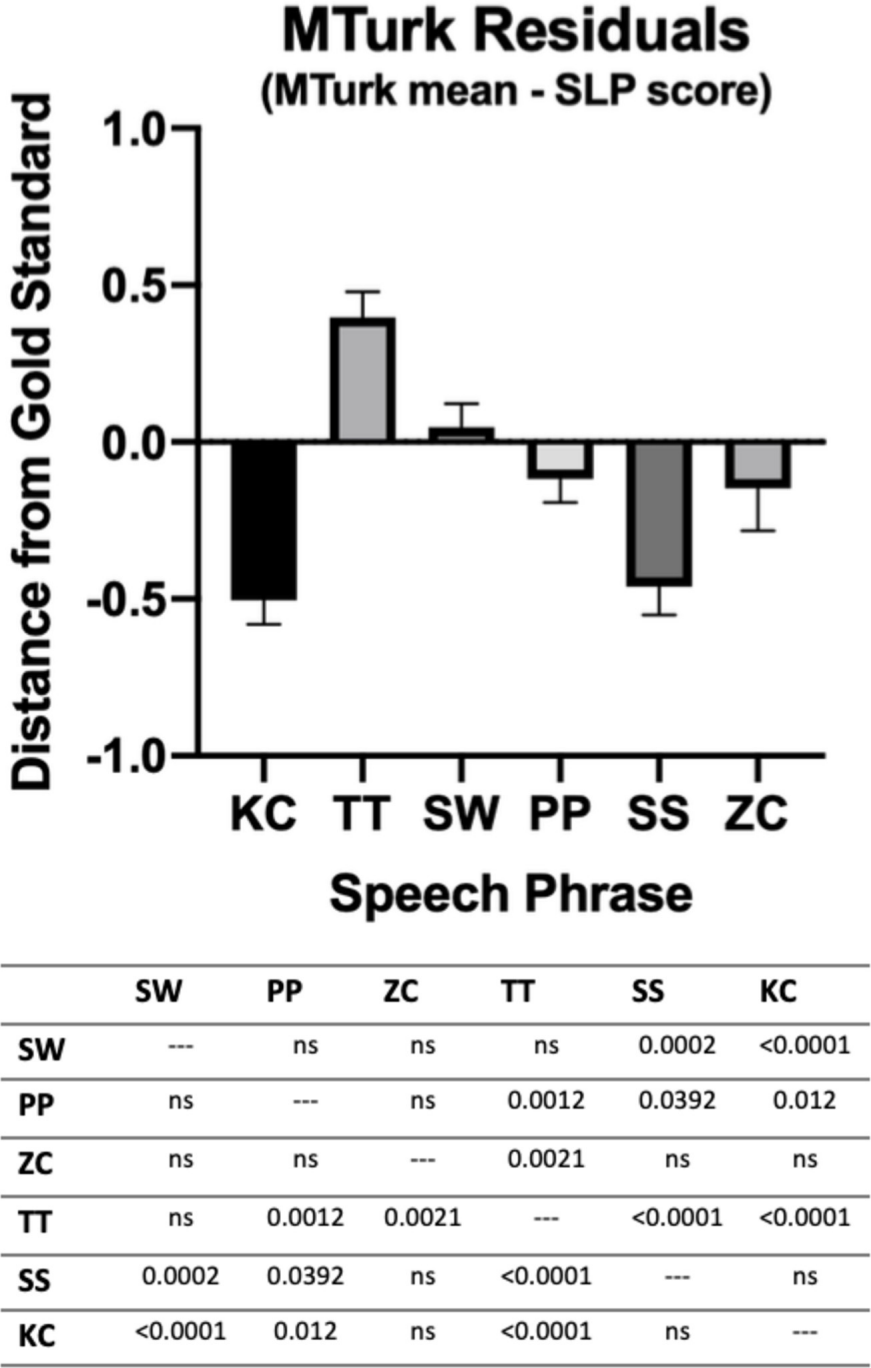
MTurk Residuals, by Speech Phrase. The mean of the residuals was calculated for each phrase (MTurk crowdsourced mean–SLP gold standard score). Mean residuals showed SW, PP, and ZC are more accurate (smaller distance from gold standard) than TT, SS, and KC (larger distance from gold standard). The column bar graphs below represent mean and standard error of the mean. Statistical significance levels from Tukey post-hoc pairwise comparisons, represented as p values, are listed in the table below the graph.

Individual phrases were then assessed for predictive value (Table 4). To determine the accuracy of each specific phrase, residuals were calculated by subtracting the SLP rating from the crowdsourced mean, with 95% confidence intervals showing overall phrase accuracy ordered SW (more accurate) ≈ PP ≈ ZC > TT ≈ SS ≈ KC (less accurate) (Fig 1). To compare each of the phrases, ANOVA with Tukey post-hoc pairwise comparisons were performed (Fig 2).

**Table 4:** Patient MTurk Ratings by Phrase. Pilot study comparing speech language pathologist (SLP) gold standard ratings to MTurk user ratings stated as means (standard error of the mean). The different phrases are represented by sentence codes KC, TT, SD, PP, SS, ZC. Blank cells indicate that the SLP did not ask the patient to speak those phrases, and therefore there was no recording of that phrase for MTurk users to rate. When an SLP provided a range of scores for a patient, such as for Patient 1, we took the mean of the range as the gold standard (e.g. a score of “2–3” was converted to 2.5).

Child	SLP Score	MTurk Overall Mean (SEM)	KC mean (SEM)	TT mean (SEM)	SW mean (SEM)	PP mean (SEM)	SS mean (SEM)	ZC mean (SEM)
P1	2–3	2.612 (0.059)	2.250 (0.114)	2.971 (0.122)	2.912 (0.116)	2.176 (0.126)	2.750 (0.152)	---
P2	3	2.654 (0.060)	2.147 (0.110)	3.324 (0.110)	3.235 (0.117)	3.485 (0.097)	1.956 (0.140)	1.779 (0.148)
P3	2	1.768 (0.070)	1.588 (0.154)	----	1.485 (0.133)	1.427 (0.144)	1.412 (0.133)	2.926 (0.135)

## Discussion

Non-stigmatizing, intelligible speech is one of the most critical outcome measures in cleft palate surgery. The cleft team has speech language pathologists (SLPs) to rate key components of speech in children with cleft palate, including hypernasality. Limited access to SLPs due to geographical and temporal factors means that patients cannot always receive SLP evaluations as often as recommended, potentially delaying speech intervention. Therefore, there is an urgent need for alternative assessment modalities that are rapid, low-cost, and widely available to help refer these children to the appropriate expert care.

Crowdsourcing continues to revolutionize clinical outcome measurements, especially for outcomes graded on the basis of social acceptability, such as speech. Our study found that online crowdsourcing of hypernasality in children with cleft palate produced ratings of hypernasality consistent with the assessment of speech experts. This held true even in the setting of a potentially limiting methodology: a short survey with the relatively challenging task of assessing hypernasality, a concept most laypeople find unfamiliar. In all three patients who had 5–6 sentence samples each, the overall mean rating of Amazon Mechanical Turk (MTurk) crowdsourced workers generated a score concordant with that of the gold-standard rating provided by our cleft clinic SLP. This indicates that MTurk ratings can accurately approximate expert ratings for hypernasality.

Our findings are particularly surprising, because the SLP expert rating (our gold standard) was a single number representing their overall impression from a clinical encounter involving many additional single word productions, sentences, rote counting, and conversational speech. Additionally, the SLP rating was assigned based on direct in-room interaction with the patient alongside visualization of the palate using clinical instruments (eg, videonasendoscopy). Despite only selecting 5–6 phrases for online assessment, this limited sample was enough to produce excellent concordance of crowdsourced layperson ratings when compared to expert SLP ratings. In addition, given the larger speech sample experienced by the expert SLP during their clinical evaluation, the SLP may be biased towards the worst sentences or to the more memorable sentences; whereas our experiment is a simple arithmetic unweighted average of 5–6 sentences.

We further delved into the potential differences in sentence accuracy, standardizing by distance from gold standard SLP speech score (i.e., average distance of 0 would mean the crowdsourced ratings were exactly the same as the SLP gold standard speech score). This is particularly relevant moving forward, as some phrases–and by extension, phonemes–appeared to be better indicators of overall speech score than others. For example, our most accurate sentence “Should I wash the dishes” (SW) emphasized the “sh” sound, suggesting that this phoneme may elicit sounds that best approximate the true degree of hypernasality, at least in the setting of crowdsourced ratings of pre-recorded audio. In contrast, the sentence “Katie likes cookies” (KC) was our relatively least accurate sentence; this may imply that the hard “k” phoneme has a lower predictive value for overall hypernasality. These are important findings, because although speech in children with cleft palate has been studied extensively in a clinical setting, it is unclear whether the same phonemes will be appropriate for assessment of hypernasality in an online setting.

Our pilot study adds to the growing body of knowledge that suggests online crowdsourcing for perceptual speech analysis provides ratings highly concordant with expert ratings [6]. This is the first study we are aware of that applies online crowdsourcing in the evaluation of hypernasal speech in children with cleft palate. It builds on previous work suggesting a role for untrained listeners in the evaluation of speech in children with cleft palate, specifically in the identification of speech hypernasality. Past studies conducted in non-online settings have found that untrained listeners are able to identify hypernasality in audio recordings of speech from children with cleft palate with a high degree of accuracy and provide ratings that are consistent with ratings of trained speech experts [8–12].

Despite the overall consistency between lay raters and experts, discrepancies emerged in some studies. For example, one study found that trained listeners such as SLPs and physicians provided more consistent ratings than their untrained counterparts, and that on average, lay raters provided higher ratings of hypernasality than their trained peers [9]. Critiques also arose regarding study methodologies, highlighting one of the major challenges in this study: identifying a standardized assessment tool that is valid among untrained listeners, and allows for direct comparison with SLP expert ratings.

Due to the presumed unfamiliarity with clinical speech variables such as “hypernasality,” many studies attempted to evaluate speech using modified scales for lay raters that applied speech descriptors (eg, “this child sounds like he/she has a cold,” speech sounds like the child is “talking through their nose,” or “sounds like puffs of air coming through the nose”) in place of validated clinical instruments used by experts [8,11,13]. While these studies may have be formatted in language more accessible to the general public, they limit the authors’ ability to make conclusions regarding consistency between layperson ratings and those of experts. Our study addressed this challenge by employing a validated speech scale used by our cleft team SLPs (the Pittsburgh Weighted Speech Score), and embedding clickable gold-standard reference samples for ratings of no hypernasality, mild hypernasality, and severe hypernasality for lay raters to reference as many times as necessary throughout the study. This approach, therefore, allowed for direct comparison between lay ratings and speech expert ratings using a validated speech scale, while also providing the lay raters examples of the disordered speech patterns they were tasked to identify.

Although lay evaluation of hypernasality appears to have a high degree of accuracy, it is important to note that some studies have found that lay ratings of other speech components may deviate from expert ratings. One study evaluating various components of speech of individuals with cleft palate found that despite similar ratings of the speech hypernasality component, untrained listeners are less adept at identifying audible nasal air emission and turbulence, two other features of speech in individuals with cleft palate, and were only able to consistently identify speech samples with relatively severe impairments[8]. Other studies have identified limitations unique to the online setting: for example, one study evaluating dysarthria in patients with Parkinson’s Disease found that lay ratings of speech volume correlated poorly with expert ratings, despite high correlation in the assessment of other speech variables [14]. These findings reinforce the importance of carefully scrutinizing study methodology and suggest that even in the case of well-designed studies, some components of speech may be more appropriately assessed in the clinical setting than in online setting, either due to the complexity of speech patterns (in the case of nasal air emission and turbulence) or the variability of sound quality in speech assessments conducted over computers (in the case of volume in the speech of patients with Parkinson’s Disease).

Identifying areas where SLPs and laypeople diverge in their perceptual speech ratings has the potential to be quite valuable, as discordance should prompt further review as to whether additional intervention is necessary. SLPs are trained to scrutinize speech for underlying pathology, but presence of pathology does not necessarily correlate with functional disability (for example, a minimal hypernasality that SLP detects, but an average layperson cannot detect). If the ultimate goal of speech is to effectively communicate with the public, then layperson perspective (including which pathologies go undetected) is not only valuable, but likely essential for patient-oriented outcome measures.

### Limitations

One limitation of this study is the sample size of three patients from our cleft center. In the future, this study can be replicated with a larger number of patients and a more diverse array of speech samples (e.g., greater variation in severity of hypernasality, regional accents, and patient ages), in order to investigate the generalizability of these results. Ratings of conversational speech, instead of isolated phrases, provide additional insight into perceived speech hypernasality, but our study attempted to show that a simpler screening tool based on shorter phrases can still have potential to provide useful information that may guide a patient to seek an expert SLP. Another limitation was the quality of speech recordings. All audio samples used in this study were extracted from videonasendoscopy recordings that were part of the patient’s medical record. The quality of these recordings was often less than ideal, sometimes with considerable white noise and audio distortion. In an effort to maintain the integrity of the initial recordings, we intentionally did not edit recordings for improved sound quality. Despite the potential for audio quality to degrade perception, layperson assessment of these recordings was highly concordant with SLP ratings. The ability of lay raters to detect hypernasality, even in the setting of limited-quality recordings, is reassuring. Moving forward, using higher quality recordings may provide additional insight and fidelity into the utility of crowdsourcing for speech outcomes in individuals with cleft palate.

Furthermore, although online lay raters may be able to identify hypernasality in speech, perceptual speech analysis alone cannot identify the root of speech disorders. Clinical ratings of speech in individuals with cleft palate traditionally combine clinical instruments (eg, videonasendoscopy) and perceptual assessments to comprehensively evaluate nasality [15], and crowdsourced ratings cannot replace this need for in-person expert clinical assessments. Our study, therefore, suggests that crowdsourcing may be an appropriate screening tool to identify hypernasality of speech, direct referrals for children in need of additional follow-up, and complement clinical ratings, but crowdsourcing alone cannot replace clinical evaluations.

### Future directions

One of the most surprising results is that the unweighted arithmetic mean of a relatively small number of sentences, approaches the accuracy of a SLP in-person clinical evaluation with many more sentences. This implies some sentences are less accurate than others, and our data suggests sentences with the “sh” phonemes may be more accurate for the assessment of hypernasality. Future directions include removing less accurate sentences from our sampling model, and evaluating whether a smaller number of highly-predictive sentences enhances the ability of untrained listeners to approximate the patient’s expert speech score.

This study sets the stage for a number of future investigations, including larger-scale multi-center studies to validate our results, studies of speech pathologies aside from hypernasality (e.g., nasal emission, nasal turbulence, hyponasality), and creation of a mobile application (app) that allows for collection of speech assessments remotely and prospectively. Remote assessment of speech, delivered through MTurk-generated perceptual speech ratings, could provide close monitoring of speech outcomes for children without ready access to SLPs. These assessments would provide non-experts, such as educators and general pediatricians, access to a screening tool for children who may need additional intervention by a qualified speech expert, facilitating timely referral and treatment. In addition, it could facilitate collaboration among SLPs in challenging cases by creating an easy system to share speech samples and garner additional speech ratings.

Online crowdsourcing has the potential to attenuate clinical barriers to care by providing rapid, low-cost assessments of speech that are concordant with gold standard measures. Discovering the most specific and accurate sentences could provide a screening tool for non-SLP clinicians and community members. Through this improved access to care, we will be able to provide better follow-up to our most vulnerable patients, including those from rural, international, or socioeconomically disadvantaged communities. Better access to diagnostic tools to facilitate earlier intervention, may improve speech outcomes and ultimately improve quality of life for children with cleft palate.

## Conclusion

Assessment of speech is a cornerstone in the post-operative care of a child born with cleft, as normal intelligible speech contributes meaningfully to a growing child’s quality of life. The results of this pilot study demonstrate that online crowdsourced ratings of hypernasal speech from children with cleft palate are highly concordant with speech expert ratings, and sets the stage for future large-scale validation studies. Furthermore, individual phrases had different crowdsourced layperson accuracies; in particular phrases with “sh” phonemes seem particularly accurate for laypeople to detect hypernasality in our online speech evaluation model. This ability to collect high-quality assessments of hypernasal speech using online crowdsourcing may provide a much-needed and exciting opportunity to extend access to care and improve patient outcomes.
